# Dysregulated gene expression predicts tumor aggressiveness in African-American prostate cancer patients

**DOI:** 10.1038/s41598-018-34637-8

**Published:** 2018-11-05

**Authors:** Hamdy E. A. Ali, Pei-Yau Lung, Andrew B. Sholl, Shaimaa A. Gad, Juan J. Bustamante, Hamed I. Ali, Johng S. Rhim, Gagan Deep, Jinfeng Zhang, Zakaria Y. Abd Elmageed

**Affiliations:** 1 Department of Pharmaceutical Sciences, Rangel College of Pharmacy, Texas A&M Health Sciences Center, Kingsville, TX USA; 2 Department of Statistics, Florida State University, Tallahassee, FL USA; 3 Departments of Pathology, Tulane University School of Medicine, New Orleans, LA USA; 4 Department of Surgery, Uniformed Services University of the Health Sciences, Bethesda, MD USA; 5 Department of Cancer Biology, Wake Forest Baptist Medical Center, Winston-Salem, NC USA; 6 Department of Radiobiological Applications, Nuclear Research Center, Atomic Energy Authority, Cairo, Egypt

## Abstract

Molecular mechanisms underlying the health disparity of prostate cancer (PCa) have not been fully determined. In this study, we applied bioinformatic approach to identify and validate dysregulated genes associated with tumor aggressiveness in African American (AA) compared to Caucasian American (CA) men with PCa. We retrieved and analyzed microarray data from 619 PCa patients, 412 AA and 207 CA, and we validated these genes in tumor tissues and cell lines by Real-Time PCR, Western blot, immunocytochemistry (ICC) and immunohistochemistry (IHC) analyses. We identified 362 differentially expressed genes in AA men and involved in regulating signaling pathways associated with tumor aggressiveness. In PCa tissues and cells, *NKX3.1, APPL2, TPD52, LTC4S, ALDH1A3* and *AMD1* transcripts were significantly upregulated (p < 0.05) compared to normal cells. IHC confirmed the overexpression of TPD52 (p = 0.0098) and LTC4S (p < 0.0005) in AA compared to CA men. ICC and Western blot analyses additionally corroborated this observation in PCa cells. These findings suggest that dysregulation of transcripts in PCa may drive the disparity of PCa outcomes and provide new insights into development of new therapeutic agents against aggressive tumors. More studies are warranted to investigate the clinical significance of these dysregulated genes in promoting the oncogenic pathways in AA men.

## Introduction

The mortality rate of prostate cancer (PCa) is 2–3 times higher in African American (AA) compared to Caucasian American (CA) men^1^. The high incidence and mortality rates of PCa among AA men are thought to be associated with genetic, lifestyle or socioeconomic-related factors^2^. Molecular mechanisms underlying these genetic discrepancies have not yet fully understood. Growing research aims to determine the contribution of these factors to such disparity among AA men. Androgen receptor (AR) signaling is among the most studied pathways in aggressive tumors of AA men^3^. Indeed, the frequency of AR mutations was higher in AA versus CA men^4^. However, the driving forces of PCa disparities are multifactorial events. For instance, the differential level of androgen metabolizing enzymes, AR-associated mRNAs, microRNAs and other genetic and epigenetic factors^5–9^ contribute to such disproportionate outcomes in AA patients compared to other races. Therefore, several studies have attempted to decipher the molecular differences between AA and CA patients with PCa^8,10,11^. Mounting evidence suggests that when other risk factors adjusted, AA men were able to develop tumors with aggressive phenotypes^12^. At higher tumor grades, PSA level and biochemical recurrence have been shown to be higher in PCa of AA men^13^. Over the last decade, a number of studies have utilized high-throughput technology including microarray for gene expression profiling as a reliable tool for biomarker discovery in PCa^14,15^. This approach empowered the discovery of TMPRSS2: ERG gene fusion, serine peptidase inhibitor, Kazal Type 1 (SPINK1), α-methylacyl-CoA racemase (AMACR) in addition to other potential candidates as diagnostic and prognostic markers of PCa^16–18^. A large number of biomarkers have been discovered to predict poor clinical outcomes in PCa patients^19–21^. For instance, six biomarkers had displayed a differential expression pattern in AA men^22^, and five PCa-associated genes have shown to be more methylated in tumor tissues procured from AA patients^23^. Additional evidence revealed that remarkable changes have occurred in epigenetic hallmarks of tumor tissues and these molecular events can be used as prognostic markers for tailored treatment of PCa patients^24^. Although microarray-based analyses have been widely used to segregate non-malignant versus malignant, low versus high tumor stages, localized versus metastatic, hormone-naïve versus castrate-resistant PCa patients, responders versus non-responders to radio- and chemotherapeutic agents, yet they do not have the ability to differentiate gene expression that can further stratify PCa patients based on their races and ethnicities.

In this study, we compared microarray data in PCa tissue specimens collected from 412 AA and 207 CA men to identify differentially expressed transcripts and their predicted signaling pathways contributing to the disparity outcomes among AA men. We then validated top listed differentially expressed genes by quantitative RT-PCR, ICC, Western blot and IHC analyses in Formalin-Fixed Paraffin-Embedded (FFPE) PCa tissue sections and cell lines established from PCa of AA and CA patients.

## Results

### Identification of differentially expressed genes in AA patients with PCa

We initiated our study by retrieving microarray data of 619 PCa patients; 412 AA and 207 CA collected from 11 data sets deposited in the Gene Expression Omnibus (GEO) database. After retrieving these data, we considered the most significant differentially expressed genes at a fold change of ≥2. Of those, 362 transcripts were differentially expressed in PCa of AA compared to CA men (Supplementary Table S1). From these listed genes, we selected the top 27 genes, which have a highly significant difference (p < 0.001) as shown in Table 1. The upregulated genes were *KLK2, COX5A, AZGP1, AMD1, ALDH1A3, MSMB, TPD52, OAT, TIMP4, APLP2, SOCS2, CD24, NKX3-1, SOD1, LTC4S, ANXA1, ACTA2* and *HIF1A* whereas downregulated ones were *F3, SHH, ADIPOQ, PTGDR, ALOX12, CNR1, FGF2, PTGES* and *LOX*. Some of these transcripts have been reported to have a potential role in cancer cell growth^25^, progression^26–28^, and angiogenesis^29^.

**Table 1:** List of differentially expressed mRNAs in African American compared to Caucasian American men with PCa.

	Gene name	Gene description	Fold change	*p*-value
1	KLK2	Kallikrein Related Peptidase 2	2.1445	8.28E-64
2	COX5 A	Cytochrome C Oxidase Subunit 5A	2.0025	1.16E-30
3	AZGP1	Alpha-2-Glycoprotein 1, Zinc-Binding	1.8598	9.16E-37
4	AMD1	Adenosylmethionine Decarboxylase 1	1.8589	7.16E-60
5	ALDH1A3	Aldehyde Dehydrogenase 1 Family Member A3	1.8498	8.73E-67
6	MSMB	Microseminoprotein Beta	1.8358	3.29E-32
7	TPD52	Tumor Protein D52	1.7500	7.71E-79
8	OAT	Ornithine Aminotransferase	1.6805	1.5652E-64
9	F3	Coagulation Factor III, Tissue Factor	1.6088	5.20E-67
10	APLP2	Amyloid Beta Precursor Like Protein 2	1.5712	1.61E-78
11	SOCS2	Suppressor Of Cytokine Signaling 2	1.5192	5.53E-56
12	CD24	CD24 Molecule	1.4710	1.82E-47
13	NKX3.1	NK3 Homeobox 1	1.4403	3.26E-36
14	SOD1	Superoxide Dismutase 1	1.4144	2.62E-54
15	LTC4S	Leukotriene C4 Synthase	1.3895	3.50E-71
16	ANXA1	Annexin A1	1.3807	5.11E-64
17	ACTA2	Alpha-Actin-2	1.3544	3.51E-47
18	HIF1A	Hypoxia Inducible Factor 1 Alpha Subunit	1.3416	1.59E-54
19	TIMP4	TIMP Metallopeptidase Inhibitor 4	−1.6343	1.74E-90
20	SHH	Sonic Hedgehog	−1.3137	2.13E-72
21	ADIPOQ	Adiponectin, C1Q And Collagen Domain Containing	−1.1756	1.03E-64
22	PTGDR	Prostaglandin D2 Receptor	−1.1428	6.23E-46
23	ALOX12	Arachidonate 12-Lipoxygenase, 12S Type	−1.0904	8.04E-53
24	CNR1	Cannabinoid Receptor 1	−1.0315	3.98E-82
25	FGF2	Fibroblast Growth Factor 2	−1.0208	3.54E-65
26	PTGES/COX2	Prostaglandin E Synthase	−1.0049	1.32E-66
27	LOX	Lysyl Oxidase	−1.0044	1.64E-81

### Differentially expressed genes are associated with different biological processes in PCa cells

The next question was how these transcripts contribute to PCa progression in AA men. First, we looked into top listed differentially expressed genes, which are involved in different biological processes to change cancer cells into more aggressive phenotypes. Our results showed that these dysregulated genes were associated with the regulation of cell proliferation, differentiation, motility, adhesion, migration, apoptosis, hormonal response, signal transduction, fatty acid synthesis and metabolism, protein transport and response to oxidative stress (Table 2). Moreover, these transcripts were localized at different cellular compartments to carry out their assigned cellular functions. Some of transcripts were localized in extracellular matrix, extravesicular bodies “exosomes” to regulate cell-cell communications, in lipid rafts, and in cytosol (Table S2). We demonstrated that these genes might be involved in turn on the oncogenic signaling to promote PCa progression and metastasis within favorable cellular compartments. This notion needs additional validation, and therefore we attempted further bioinformatic analyses to support these findings.

**Table 2:** List of genes contributing to different cellular biological processes in PCa.

Biological component	Gene symbol	Parents Identifier
Regulation of apoptotic process	CD24, CDKN1B, CLU, CNR1, CTNNB1, CYP1B1, EGFR, F3, ALDH1A3, FLNA, ALOX12, GREM1, ANXA1, HIF1A, GADD45B, NKX3-1, PTGS2, SHH, SOD1, SOCS2, ADIPOQ	GO:0042981
Response to steroid hormone	CD24, CDKN1B, CTNNB1, EGFR, F3, FOS, ANXA1, HIF1A, LOX, NKX3-1, PTGS2, SREBF1, SOCS2, ADIPOQ	GO:0048545
Fatty acid biosynthetic process	ALOX12, ANXA1, PTGS2, FASN, LTC4S	
Lipid metabolic process	CLU, CNR1, COMT, CYP1B1, EGFR, FASN, ALDH1A3, FGF2, ALOX12, ANXA1, IMPA1, LTC4S, PTGS2, SHH, SOD1, SREBF1, ADIPOQ, PTGES	GO:0006629
Response to estradiol	CDKN1B, CTNNB1, EGFR, F3, ANXA1, HIF1A, PTGS2, SOCS2	GO:0032355
Regulation of cell proliferation	CD24, CDKN1B, CLU, COMT, CTNNB1, CYP1B1, EGFR, F3, FGF2, ALOX12, GREM1, ANXA1, HIF1A, NKX3-1, AZGP1, PTGS2, SHH, ADIPOQ, PTGES	GO:0042127
Regulation of cell motility	CYP1B1, EGFR, F3, FGF2, ALOX12, ANXA1, PTGS2, SHH, FLNA, ADIPOQ, HIF1A, MYLK, GREM1	GO:2000145
Positive regulation of cell differentiation	CD24, CDKN1B, CLU, CTNNB1, EGFR, F3, ALOX12, GREM1, ANXA1, HIF1A, NKX3-1, PTGS2, SHH, FGF2	GO:0045597
Regulation of cell migration	CYP1B1, EGFR, F3, FGF2, FLNA, ALOX12, GREM1, ANXA1, HIF1A, MYLK, PTGS2, SHH, ADIPOQ	GO:0030334
Regulation of angiogenesis	CTNNB1, CYP1B1, F3, FGF2, ALOX12, GREM1, HIF1A, PTGS2	GO:0045765
Positive regulation of signal transduction	CD24, CLU, CTNNB1, CYP1B1, EGFR, F3, FGF2, FLNA, GREM1, HIF1A, GADD45B, NKX3-1, PTGS2, SHH, SOD1, SOCS2, ADIPOQ	GO:0009967
Regulation of protein transport	CNR1, EGFR, FLNA, GREM1, ANXA1, HIF1A, OAZ2, PTGS2, SHH, SREBF1, ADIPOQ, CDH1	GO:0051223
Regulation of cell adhesion	CD24, CTNNB1, CYP1B1, FLNA, ALOX12, GREM1, ANXA1, SHH, SOD1, ADIPOQ, CDH1	GO:0030155
Cellular response to reactive oxygen species	CYP1B1, F3, FOS, ANXA1, HIF1A, SOD1	GO:0034614
Enzyme linked receptor protein signaling pathway	CTNNB1, EGFR, F3, FGF2, CYFIP1, FOS, GREM1, HIF1A, NKX3-1, SHH, SREBF1, SOCS2, ADIPOQ	GO:0007167
Transmembrane receptor protein tyrosine kinase signaling pathway	CTNNB1, EGFR, F3, FGF2, CYFIP1, GREM1, HIF1A, NKX3-1, SREBF1, SOCS2, ADIPOQ	GO:0007169

### Dysregulated signaling pathways and their correlation to clinical outcomes

**Figure 1:**
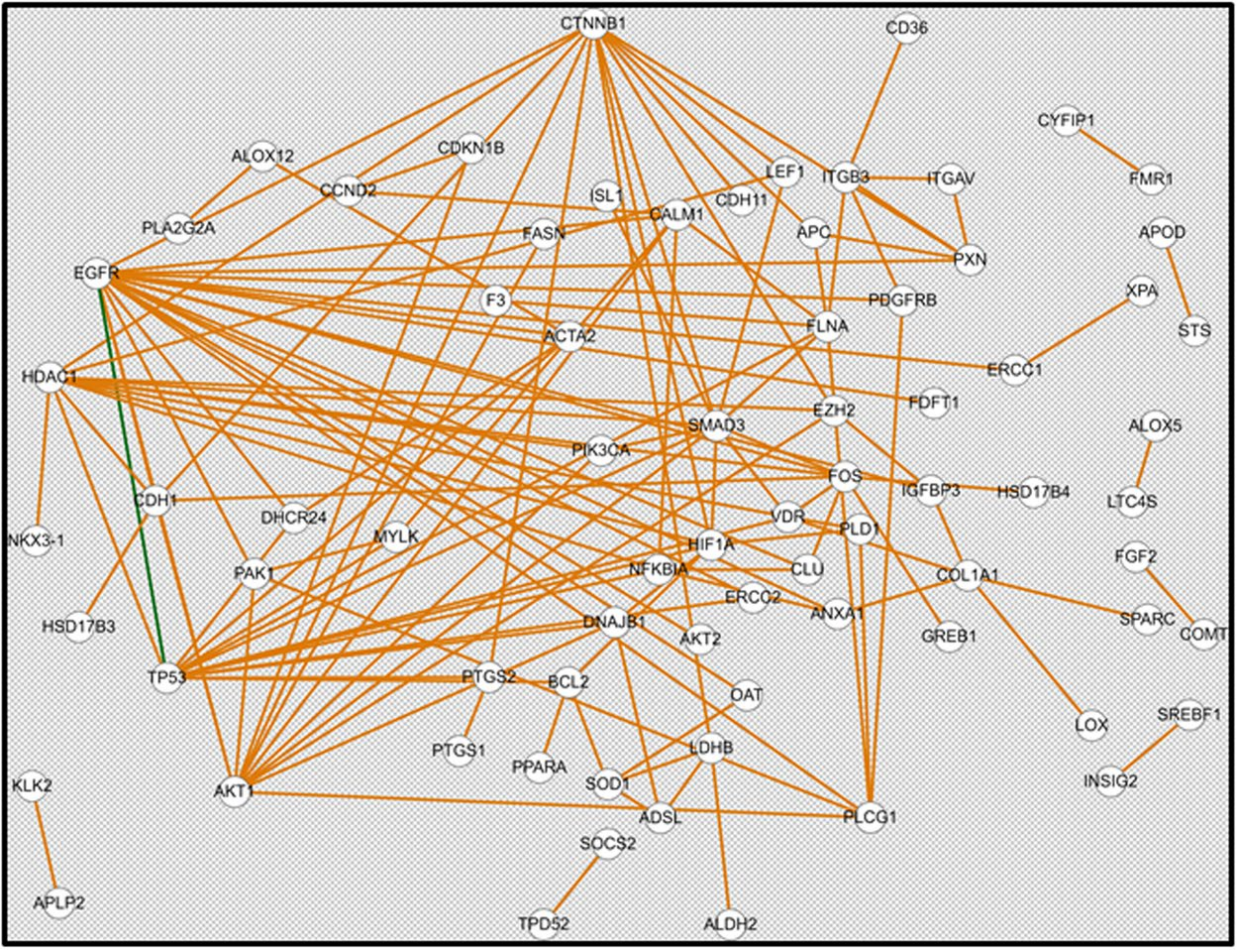
Predictive gene network constructed from differentially expressed genes. Predictive gene network for 121 differentially expressed genes at a fold change cut-off >0.7 in PCa of AA versus CA patients.

Perceptibly, our goal here was to dissect the different signaling pathways in which these dysregulated genes are involved. The humanmine.org bioinformatic software was used to identify dysregulated pathways (Fig. 1). Differentially expressed genes whose fold change value is greater than cut-off value of 0.7 were used as input. Our results showed that these genes are involved in multiple pathways of cancer, prostate cancer, focal adhesion, lipid metabolism, constitutive PI3K/AKT signaling, EGFR, PDGF, FGFR, ERBB2/DAP12 and MAPK signaling pathways (depicted in Table S3). We further investigated the association of these genes with clinical outcomes in PCa patients including age at diagnosis, pathologic grading, residual tumor, number of lymph nodes, PSA level and Gleason score as shown in supplementary Table S4.

### Validation of selected differentially expressed genes in PCa cells

**Figure 2:**
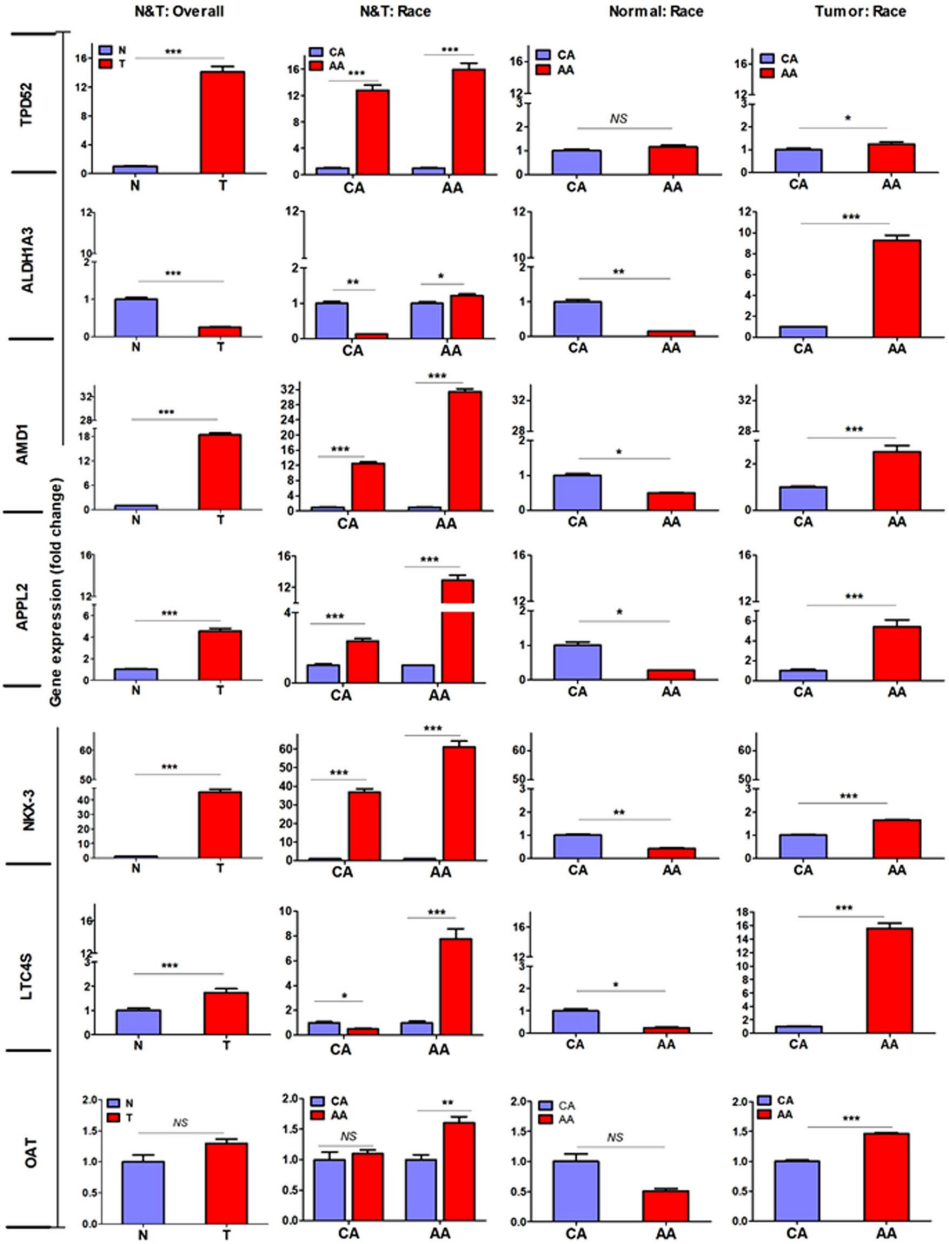
Differential gene expressions in PCa cells. RNA was extracted from PCa cells of AA and CA origin in addition to the normal cells (AA & CA). Quantitative RT-PCR analysis was performed to validate the expression of dysregulated genes in PCa cells. Fold changes of target genes were normalized against β-actin and 5S rRNA. *, **, **Depicts significance at p < 0.05, P < 0.01 and p < 0.001, respectively. NS: non-significant differences.

**Figure 3:**
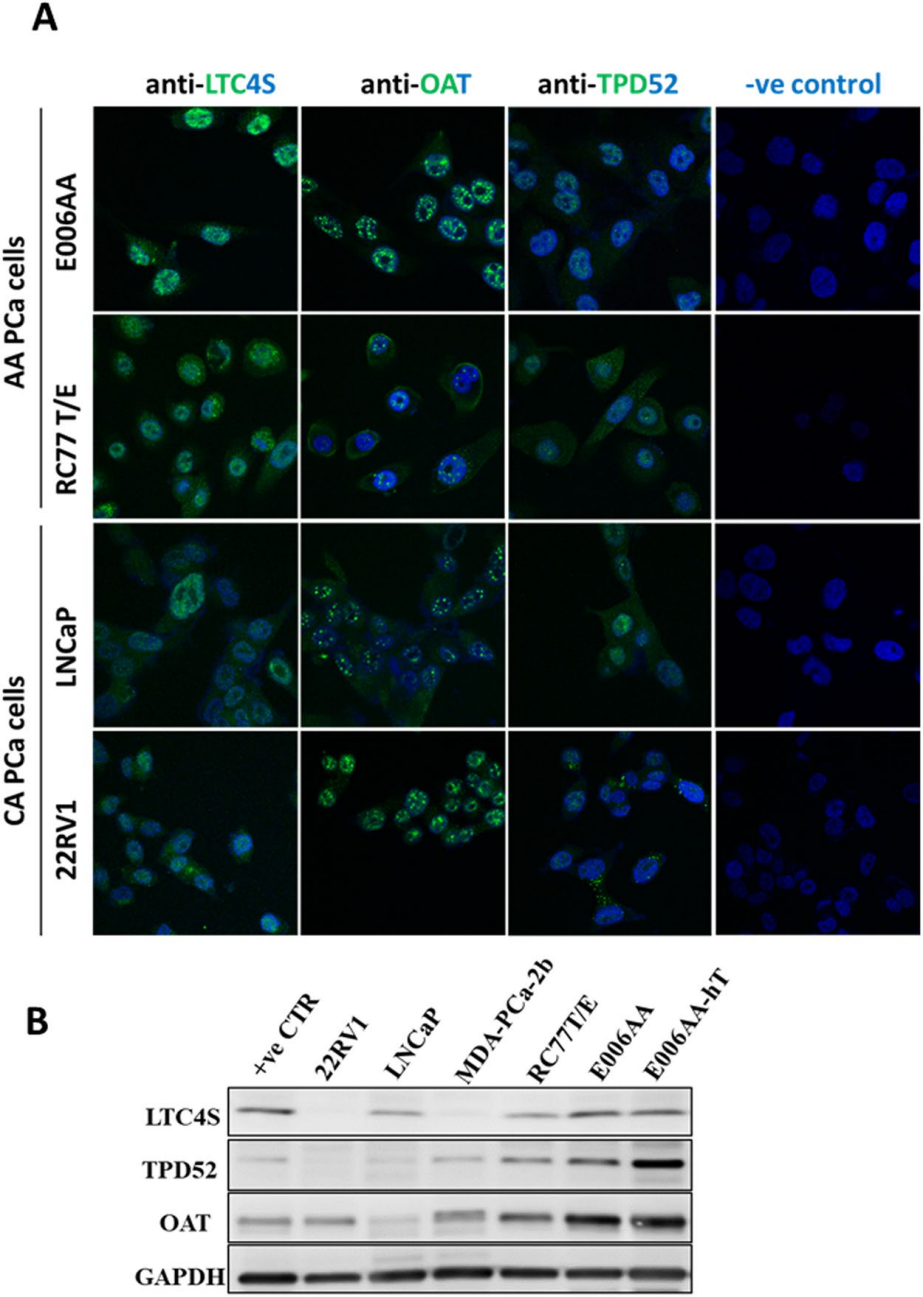
Protein expression of LTC4S, TPD52 and OAT in PCa cells. (**A**) PCa of AA origin (E006AA, RC77T/E) and CA origin (LNCaP & 22RV1) cells were cultured in a complete medium, fixed with 4% paraformaldehyde and followed by overnight incubation with anti-LTC4S, anti-TPD52 and anti-OAT antibodies at 4 °C. After washing, cells were incubated with secondary Alexa Fluor 488 antibody for 1 hour. After a series of washing, cells were mounted with mounting medium and DAPI. Developed protein signals were visualized by confocal fluorescence microscopy. Magnification was 600x. (**B**) 22RV1, LNCaP, MDA-PCa-2b, RC77T/E, E006AA and E006AA-hT in addition to breast cancer MCF7 cells (as a positive control) were cultured in the designated medium. Protein lysate was extracted from PCa cells and Western blot analysis was performed using the indicated antibodies. GAPDH was used as an internal loading control. Experiments were repeated at least twice.

The critical step in our study was to validate the expression of these candidate genes on mRNA and protein levels. We initiated our experiments by examining the gene expression by qPCR analysis using a large panel of PCa cells established from PCa patients of known AA and CA origin. We utilized both immortalized, non-tumorigenic RWPE-1 (CA-origin) and primary non-tumorigenic RC77N/E (AA-origin) as prostate epithelial control cells, LNCaP, 22RV1, DU-145 and PC-3 as PCa cells of CA-origin, and MDA-PCa-2b, RC77T/E, E006-AA and E006-AA-ht as PCa cells of AA-origin. Data from qPCR analysis demonstrated that *APPL2, AMD1, NKX3.1, LTC4S*, and *TPD52* were significantly upregulated (p < 0.001), *ALDH1A3* was downregulated (p < 0.001) while OAT did not show any significant difference in PCa versus normal cells (Fig. 2). A statistical significant difference (*p* < 0.001) was observed for each of *APPL2*, *AMD1, LTC4S*, *OAT*, NKX3.1, ALDH1A3, and *TPD52* (*p* ≤ 0.05) when PCa of AA-origin compared to PCa of CA-origin cells as shown in Fig. 2. Expression patterns of selected genes in PCa of AA and CA cell lines was confirmed on protein level by immunofluorescence and Western blot analyses for LTC4S, TPD52 and OAT (Fig. 3A,B). These proteins had different pattern of nuclear and cytoplasmic staining in AA and CA PCa cells. However, nuclear staining was mostly observed in E006AA cells (Fig. 3A) but it needs further study to determine whether cellular compartments have any role in race-associated protein trafficking in tumor cells. Immunoblots shown in Fig. 3B were reconstructed from the original immunoblots represented in supplementary Fig. S1.

### Validation of selected differentially expressed genes in human PCa FFPE tissues

**Figure 4:**
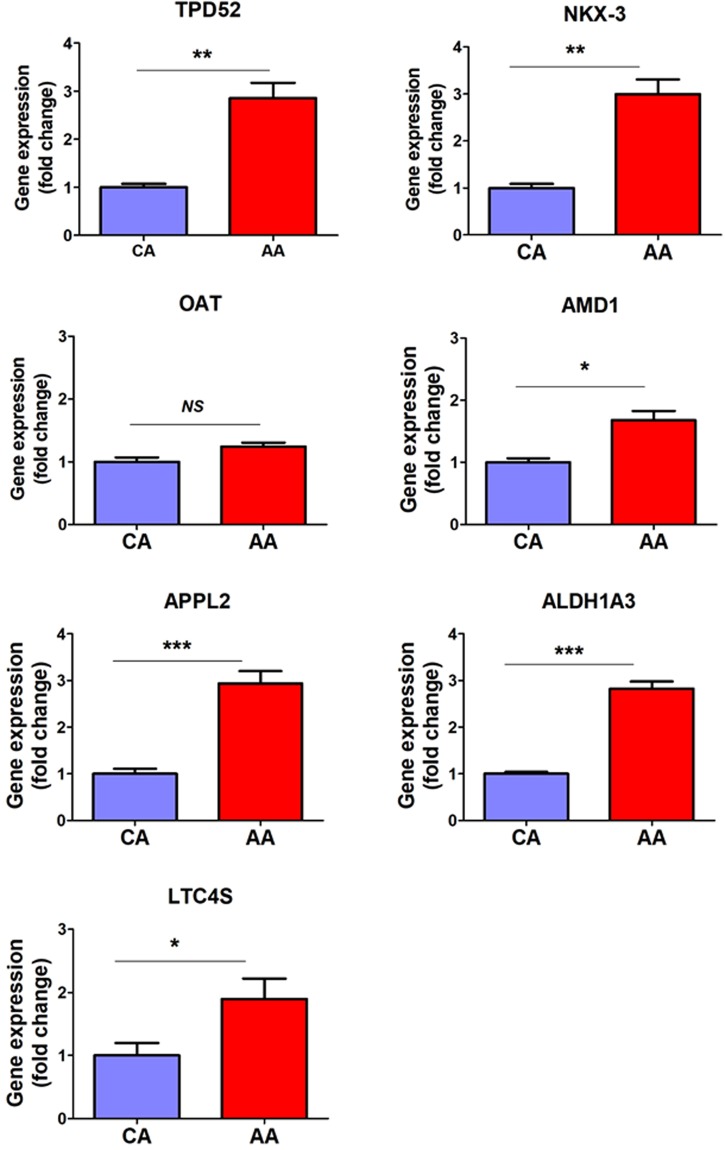
Differential gene expressions in human formalin-fixed paraffin-embedded tissue specimens collected from African American and Caucasian American men with PCa. RNA was extracted from FFPE tissue specimens collected from AA (n = 19) and CA (n = 20), and gene expression analyses were performed by qPCR. The fold change of target genes was normalized with β-actin and 5S rRNA. *, **, **depicts significance at p < 0.05, P < 0.01 and p < 0.001, respectively. NS: non-significant differences.

**Figure 5:**
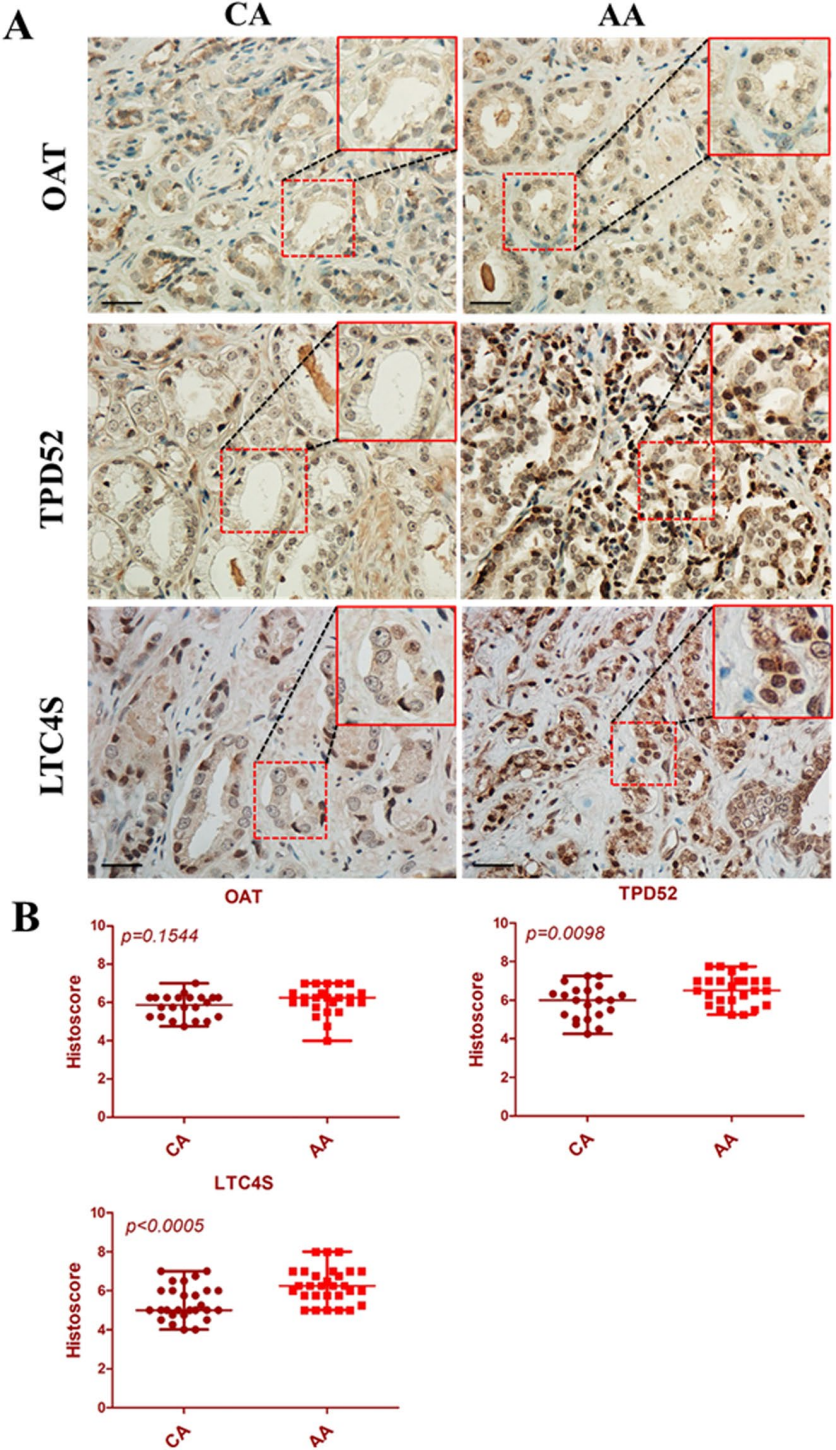
Immunostaining of human FFPE tissue sections of PCa. Tissue sections were stained with anti-TPD52, anti-OAT, and anti-LTC4S antibodies in AA (n = 29) and CA (n = 27) specimens (**A**) and the signal was developed by ABC kit as indicated. The protein localization and its intensity were blindly assessed by a cytopathologist (ABS) and semiquantitatively represented in scatter plot (**B**). The p-value for each protein was presented on each scatter plot. Scale bar was 20 µm.

Considering relative limitations of PCa cell lines, we examined the pattern of these transcripts in FFPE PCa tissues collected from 39 AA and CA patients. Before initiating this study, we stained these tissue sections with H&E followed by microscopic examination to determine the ratio of tumor to normal cells for each case and we only selected tissue blocks that contained more than 50% tumor cells. Our results revealed that transcripts of *TPD52, NKX3.1, LTC4S, APPL2, ALDH1A3*, and *AMD1* were significantly upregulated (p < 0.05) in tissues procured from AA compared to CA PCa patients as illustrated in Fig. 4. However, *OAT* did not showed any significant differences. We then correlated dysregulated genes in tumor tissues with clinical outcomes in PCa patients. As shown in Table 3, the levels of gene expression (median ΔCT) were used to stratify PCa patients into two groups; low and a high expression groups. The percentage of positive cores was significantly elevated in the high expression group of *APPL2*, *AMD1* and *TPD52* (p < 0.05). Likewise, the percentage of tumor involvement in the prostate gland showed a significant elevation in high expression group of *ALDH1A* (p = 0.038) and *APPL2* (p = 0.054) compared to its counterpart group. The high expression of *ALDH*, *AMD1* and *OAT* was correlated with prostate volume. To this extent, we validated the data from the bioinformatic analysis in human FFPE PCa tissues on an mRNA level, however, the protein expression in these tissues are necessary to evaluate the expression of these candidate genes in PCa tissues. We stained PCa tissue sections collected from 56 AA and CA patients with antibodies raised against OAT, TPD52 and LTC4S. In accordance with above-mentioned data, TPD52 (p = 0.0098) and LTC4S (p < 0.0005) showed higher protein expression in AA versus CA tissue sections; however, there was no significant change observed in OAT expression (p = 0.15544) as shown in Fig. 5A,B.

**Table 3:** Association of dysregulated genes with clinical outcomes in PCa of AA compared to CA patients.

	Age	PSA	Prostate Volume	T stage	% Positive Cores	% Involvement
APPL2	0.253	0.258	0.182	0.775	0.007*	0.054
ALDH1A3	0.156	0.388	0.049*	0.545	0.074	0.038*
AMD1	0.098	0.470	0.041*	0.583	0.024*	0.430
LTC4S	0.219	0.352	0.192	0.871	0.475	0.126
NKX3.1	0.081	0.374	0.207	0.626	0.199	0.103
OAT	0.077	0.497	0.022*	0.301	0.813	0.867
TPD52	0.205	0.383	0.140	0.486	0.025*	0.581

PSA: Prostate-specific antigen. Data represents p-values calculated by Mann-Whitney to correlate gene expression levels (median ΔCT) with clinical characteristics of PCa patients. *Depicts statistical significance at p < 0.05.

## Discussion

In this report, we established a sharp contrast between the expression pattern of AA and CA PCa patients by analyzing microarray data from GEO database. Gene ontology and bioinformatics analyses revealed that these genes have a potential role in PCa aggressiveness in AA men by altering cellular signaling pathways in favor of tumor cells. We were able to validate this race-based contrast in the expression pattern on RNA and protein levels in PCa FFPE tissues and cell lines. Our bioinformatic analysis identified unique genes associated with multiple biological processes and cellular trafficking in aggressive tumor cells. These include dysregulated genes, which contribute to the response to steroid hormones, fatty acid biosynthesis, regulation of cell proliferation, adhesion and motility, regulation of cell migration, and protein kinase signaling pathway. These genes including but not limited to NKX3.1, SHH, EGFR, HIF1A, CTNNB1, FASN and others. Prior studies suggested that the function of NKX3.1 is frequently lost in castrate-resistant PCa and associated with genomic instability and biochemical relapse-free when combined with c-MYC^30^. Using genetically engineered mouse model, NKX3.1-PTEN mutant mice developed androgen-independent aggressive tumors^31^. The second gene linked to aggressive PCa phenotype is the Sonic Hedgehog (SHH). SHH pathway is involved in PCa angiogenesis, metastasis and development of drug resistance^32^. Another evidence is that SHH-Gli1 axis is associated with transforming malignant PCa stem cells into metastatic-like cells^33^. In the same context, epidermal growth factor receptor (EGFR) is another dysregulated gene whose signaling pathway is well known to be involved in cell proliferation, migration, adhesion and its overexpression is correlated with poor prognosis^34^. Hypoxia-inducible factor 1 (HIF-1), as one of our candidates, facilitates tumor cells to adapt for hypoxic conditions by regulating genes associated with hormone-refractory progression, angiogenesis, metastasis, and therapeutic resistance^35^. Additionally, the expression of β-catenin was higher in PCa and associated with disease progression^36^. Indeed, adaptive metabolic pathways and their linked lipid rafts are important step in the process of metastasis. For instance, overexpression of fatty-acid synthase (FSAN) is associated with PCa progression and metastasis^37^. In response to steroid hormones, we previously reported that circulating estrogen and expression of ERβ were substantially higher in PCa tissues of AA men^38^. In addition to the above-mentioned dysregulated genes, we reported other novel genes where their roles in PCa aggressiveness need more investigations.

We validated top listed genes in PCa cells and found that *APPL2, AMD1, ALDH1A3, LTC4S, OAT* and *TPD52* were upregulated in PCa of AA compared to CA cells. On tissue level, these genes were upregulated in FFPE tissues of AA and were significantly correlated with prostate volume, percentage of positive cores and percentage of tumor involvement. In this study, we identified TPD52, AMD1 and LTC4S in addition to other dysregulated genes as potential candidates that might be associated with PCa aggressiveness among AA men. Other previous studies have supported our findings of the strong link between these candidate genes and tumor aggressiveness. For example, tumor protein 52 (TPD52) is an oncogenic protein expressed in malignant tissues including PCa^27,39,40^. The overexpression of TPD52 in LNCaP cells induced cell growth, colonogenic growth, migration and Akt activity^41^. Equally important, overexpression of S-adenosylmethionine decarboxylase 1 (AMD1) promotes tumor growth by increasing biosynthesis of polyamines, and foci formation anchorage-independent cell growth^42^. Chronic inflammations in the prostate gland account for ~20% of carcinogenesis of PCa^43^, and these inflammatory responses predicting tumor aggressiveness and poor clinical outcomes^44,45^. Interestingly, the prostate gland luminal epithelial layer adjacent to infiltrating immune cells shows atrophic appearance^46^. Luminal cells with low expression of CD38 can transform into PCa cells in the presence of oncogenic inducers^44^. Mounting evidence shows that arachidonic acid pathway contributes to PCa development and progression. For example, omega-6 polyunsaturated fatty acid arachidonic acid promotes the migratory PCa cells to the stroma of the bone marrow^47^. A close look into the top dysregulated genes in AA suggests the role of arachidonic pathway in aggressive tumors. As such, one of the top listed genes in AA is LTC4S where its role in inflammatory responses is well known^48–50^, however, its role has not yet investigated in PCa aggressiveness. While our results demonstrated that ornithine aminotransferase (OAT) could not segregate AA from CA men, it is associated with AR signaling pathway. OAT, an enzyme required for the metabolism of polyamines, is AR-target gene functions in a ligand-independent fashion^26^. Disturbance of cellular localization of key proteins involved in regular functions of cells may change the activities of these proteins. For example, Gu *et al*. reported that benign prostate epithelium had nuclear localization of PRMT5 while PCa tissues had cytoplasmic localization, which suggests the role of cellular localization of PRMT5 in cell growth and tumorigenesis of PCa^51^. In another study by Scher *et al*., the localization of AR-V7 in the nucleus is critical for selecting treatment options offered to PCa patients with metastatic castration-resistant^52^. In the light of this evidence, we observed different cellular localization of OAT, LTC4S and TPD52 in PCa cells procured from AA and CA, which may imply a possible role in tumor aggressiveness in AA but it needs further studies. The strength of our study includes the bioinformatics analysis performed on a large number of PCa of AA patients followed by prediction of the oncogenic pathways of dysregulated genes, and their correlation with clinical outcomes in AA men. One of the limitations of the study is the use of tissue specimens collected from one cohort in validation steps; however, we validated these genes on RNA and protein levels in a number of FFPE PCa tissues and cells collected from AA and CA patients. Therefore, our findings are presenting molecular foundations by which we determined the clinical significance of these dysregulated genes in segregation of PCa patients according to their race and their association with poor clinical outcomes in AA men. More studies are warranted to investigate how these genes promote oncogenic signaling pathways and drive tumor cells towards aggressiveness in AA men. In conclusion, our findings suggest that dysregulation of transcripts in large number of PCa of AA compared to CA men may explain the aggressive behavior of PCa. Our data provide new insights into novel as well as known candidates involved in PCa disparity and might be of clinical significance as prognostic markers or therapeutic targets in AA men at advanced stages of the disease.

## Materials and Methods

### Data collection

Expression microarray data of 619 PCa patients were collected from 11 data sets in the GEO database including 412 AA and 207 CA patients. The raw gene expression counts were normalized by linear normalization according to the following equation:

$${r}_{i,j}=\frac{{x}_{i,j}}{\sum _{j=1}^{p}{x}_{i,j}}\times {10}^{6},i=1,\ldots ,n\,{\rm{samples}};j=1,\ldots ,p\,{\rm{genes}}$$

where *x* denotes the gene expression count, and *r* denotes the read per million.

To perform differential gene expression analysis for AA and CA, two-sample *t*-test with procedures in SAM (Significance Analysis of Microarrays) was applied^53^. To identify the differentially expressed genes for other clinical features, we collected the correlation analysis results from Broad Institute^54^, and adjusted the *p*-values using BH method based on the number of genes used in this study.

### False discovery rates (FDR)

Adjusted *p*-value ≤ 0.05 and fold change of ≥2 were used for reporting significantly differentially expressed genes (unless otherwise noted) to reduce the number of false positives. If possible, the Benjamini-Hochberg procedure was used to control for FDR and is reported in the results^55^. Benjamini-Hochberg is used in DESeq2 output by default. *P*-values that have been adjusted are denoted to as adjusted *P*-values.

### Pathway analysis and visualization

The differentially regulated pathways were generated from humanmine.org using the differentially expressed genes identified in gene level analysis^56^. We selected the differentially regulated pathways with adjusted *p*-values of less than 0.05. For pathway visualization, Pathview package in R was adopted.

### Cell culture

The human PCa cell lines LNCaP, C4-2B, DU145, PC-3, and MDA-PCa-2b as well as RWPE-1 cells, a non-tumorigenic immortalized human prostatic epithelial cell line derived from CA donor, were obtained from American type culture collection (ATCC, Manassas, VA). PCa E006AA and E006AA-hT cells were kindly provided by Dr. M Saleem (The University of Minnesota) and maintained as described^57^. Cells were cultured and maintained in DMEM medium containing 10% Fetal Bovine Serum (Gibco, Carlsbad, CA, USA) and 1% Penicillin/Streptomycin. RC77T/E AA PCa cells and their matched immortalized RC77N/E normal prostate cells were maintained as described^58^. RWPE-1, RC77T/E and RC77N/E cells were grown in keratinocyte serum-free media supplemented with bovine pituitary extract and epidermal growth factor following the manufacturer’s protocol (Life Technologies Corp., Grand Island, NY). MDA-PCa-2b cells were grown in HPC1 medium (Athena Environmental Sciences Inc., Baltimore, MD) supplemented with 20% FBS and 50 µg/mL G418. Cells were maintained at 37 °C and a humidified incubator containing 5% CO_2_. Cells were authenticated and confirmed that they were free from mycoplasma.

### PCa tissues, RNA extraction and Real-Time PCR analysis

Formalin-fixed paraffin-embedded (FFPE) archival PCa tissues were obtained from the Louisiana Cancer Research Center (LCRC) Biospecimen Core, New Orleans, LA. All research work was performed in accordance with relevant guidelines of a protocol approved by the Institutional Review Board (IRB) from Tulane University School of Medicine, New Orleans, LA. Informed consent was obtained from all PCa patients involved in this study. Total RNA extracted from FFPE tissues of PCa patients using RNeasy FFPE kit according to the manufacturer’s protocol (Qiagen; Germantown, MD). Briefly, four freshly cut 10 µm-thick FFPE tissue sections were used per each sample, deparaffinized with heptane and methanol, air-dried and digested with proteinase K, and incubated in high temperature (80 °C) for crosslinking reversal. The tissue lysate treated with RNase-free DNase to eliminate any DNA contamination. RNA precipitated with 100% ethanol, applied to spin column, washed and eluted in 30 µl of RNase free water. Total RNA from PCa cells was extracted using the Trizol reagent according to the manufacturer’s protocol (Invitrogen Corp., Carlsbad, CA, USA). cDNA was prepared using M-MuLV Reverse Transcriptase and random primer mix according to the standard protocol (New England Biolabs, Ipswich, MA). qPCR was performed using SYBR Green master mix (Bio-Rad, Hercules, CA, USA) on a Bio-Rad CFX96 detection system. PCR products were run on agarose gel to assure the specificity of each primer. The list of primer sets used in this study was described in supplementary Table S5. The fold change of gene expression was calculated relative to β-actin and 5S rRNA by comparing Ct method as described^59^.

### Western blot analysis

Western blot analysis was performed as previously described^60^. Briefly, about 20 µg whole protein lysate was loaded onto a 4–20% SDS-PAGE gel (Bio-Rad, Hercules, CA) under reducing conditions. The fractionated proteins were transferred onto a nitrocellulose membrane (Bio-Rad, Hercules, CA), which was subsequently blocked with 5% bovine serum albumin for 1 hour. The membranes were incubated overnight at 4 °C with antibodies raised against OAT, TPD52, and LTC4S (Biorbyt, San Francesco, CA). Anti-GAPDH was used as an internal protein loading control (Santa Cruz Biotechnology, Dallas, TX). The membranes were washed thoroughly in washing buffer and incubated with the proper secondary antibodies for 1 hours at room temperature. After another series of washing, the membranes were developed and visualized by Odyssey^®^ Fc Imager and C-Digit Blot Scanner (LI-COR, Lincoln, NE).

### Immunofluorescence

Immunofluorescence was carried out as previously described^60^. PCa cells were cultured in chamber slides (Fisher Scientific, Hampton, NH), washed and fixed in 4% paraformaldehyde. After another series of washing, cells were permeabilized and blocked with 2% BSA in TBST buffer. Cells were incubated overnight at 4 °C with primary antibodies as indicated. Next, cells were incubated with Alexa Fluor® 488 secondary antibody, then stained with 4′ 6′-diamindino-2-phenylindole (DAPI) and mounting medium (Vector Laboratories, Burlingame, CA). Images were acquired under Nikon D-ECLIPSE C1si spectral laser-scanning confocal (Nikon Instruments, Melville, NY).

### Immunohistochemistry

Immunohistochemical (IHC) staining with anti-OAT, anti-TPD52 and anti-LTC4S antibodies (Biorbyt, San Francesco, CA) was performed according to our reported protocol^60^. Briefly, tissue sections were de-waxed in xylene and rehydrated in descending series of ethyl alcohol. Tissue slides were then heated in 0.01 M citrate buffer pH 6.0 (Newcomer Supply, Maddison, WI) for 20 min in a steam cooker. The sections were immersed in 3% hydrogen peroxide for 10 min to block endogenous peroxidase activity. The slides were incubated overnight with primary antibodies at 4 °C. Bound antibody was detected by avidin-biotin complex peroxidase method using an ABC Elite Kit (Vector, Burlingame, CA, USA) with 3,3′-diaminobenzidine (DAP) as a chromogen. Tissues were counterstained with Mayer’s hematoxylin solution and lithium carbonate as a bluing agent (Newcomer Supply, Maddison, WI). The immunostaining signals were visualized and captured using Eclipse 80i microscope (Nikon Instruments, Melville, NY). The intensity of the developed staining was blindly assessed by a cytopathologist (ABS). The histoscore was calculated as we described^60^.

### Statistical analysis

Data were presented as mean ± standard error of mean. Comparison between experimental and their control counterparts were performed by applying Mann-Whitney test and Welch-corrected unpaired t-test using GraphPad Prism 7.0 (GraphPad Software, Inc., La Jolla, CA). An adjusted p-value of less than 0.05 was considered significant.

## Electronic supplementary material

Supplementary information

## Data Availability

The microarray data and other associated generated data of the current study are available on request.
