# Supplementary material for: Dysregulated gene expression predicts tumor aggressiveness in African-American prostate cancer patients
Source: Sci Rep. 2018 Nov 5;8:16335. doi: 10.1038/s41598-018-34637-8 (PMC6218553; doi:10.1038/s41598-018-34637-8)

Supplementary Information

Dysregulated gene expression predicts tumor aggressiveness in African-American prostate cancer patients

Hamdy E.A. Ali[1], Pei-Yau Lung[2], Andrew B. Sholl[3], Shaimaa A. Gad[1], Juan J. Bustamante[1], Hamed I. Ali[1], Johng S. Rhim[4], Gagan Deep[5], Jinfeng Zhang[2], Zakaria Y. Abd Elmageed[1]

Department of Pharmaceutical Sciences[1], Rangel College of Pharmacy, Texas A&M Health Sciences Center, College Station, TX; Department of Statistics[2], Florida State University, FL; Departments of Pathology[3], Tulane University School of Medicine, New Orleans, LA; Department of Surgery[4], Uniformed Services University of the Health Sciences, Bethesda, MD; Department of Cancer Biology[5], Wake Forest Baptist Medical Center, Winston-Salem, North Carolina, USA.

Supplementary Table S1. List of differentially expressed genes in AA vs. CA PCa patients using a cut-off value of 0.7

Gene.ID	p-value	Fold change abs
KLK2	8.28E-64	2.144526978
COX5A	1.16E-30	2.002497329
AZGP1	9.16E-37	1.859830545
AMD1	7.16E-60	1.85888259
ALDH1A3	8.73E-67	1.849769455
MSMB	3.29E-32	1.835768182
TPD52	7.71E-79	1.749998337
OAT	1.5652E-64	1.680530794
TIMP4	1.74E-90	1.634342728
F3	5.20E-67	1.608769498
APLP2	1.61E-78	1.571195789
SOCS2	5.53E-56	1.519196821
CD24	1.82E-47	1.47095648
NKX3-1	3.27606E-36	1.440336555
SOD1	2.62E-54	1.414377983
LTC4S	3.50E-71	1.389522825
ANXA1	5.11E-64	1.380664724
ACTA2	3.51E-47	1.354434009
HIF1A	1.59E-54	1.341581118
SHH	2.13E-72	1.313683969
FOS	3.25E-35	1.2599163
EGFR	2.17E-39	1.23708082
FASN	1.60E-66	1.225507581
GYG1	9.11E-83	1.216057587
CTNNB1	2.99E-44	1.17623897
ADIPOQ	1.03E-64	1.175573103
GREB1	1.01E-65	1.166381175
OAZ2	1.45E-64	1.157729868
PTGDR	6.23E-46	1.142835711
GADD45B	4.16E-37	1.141654102
MYLK	2.30E-48	1.13605219
PTGS2	8.89E-36	1.125932834
GREM1	2.34E-42	1.108720887
ALOX12	8.04E-53	1.090356453
COMT	7.12E-82	1.083529729
CYP1B1	1.89E-58	1.08274872

SREBF1	7.31E-26	1.070315082
CLU	2.31E-47	1.048953619
DES	3.93E-34	1.043709986
CDH1	4.86E-46	1.041299455
CNR1	3.98E-82	1.031466083
IMPA1	3.37E-20	1.027506872
FLNA	2.41E-41	1.022535028
FGF2	3.54E-65	1.020790345
LDHB	1.57E-37	1.012453079
CDKN1B	6.35E-28	1.011885765
CYFIP1	5.07E-53	1.011624587
PTGES	1.32E-66	1.004919989
LOX	1.64E-81	1.004438377
FDFT1	6.87E-57	0.994295102
ADSL	2.14E-62	0.992248967
EDNRA	7.74E-44	0.985618355
PLA2G2A	3.95E-19	0.98513494
ERCC2	4.57E-77	0.975408395
ERCC1	8.15E-52	0.966264316
VDR	1.74E-20	0.960630748
SMAD3	2.33E-24	0.946722665
CLDN5	2.16E-56	0.945275512
PLAT	5.53E-23	0.935092731
PIK3CA	3.52E-64	0.92424298
MMP9	1.81E-43	0.92188515
PXN	3.67E-13	0.914278404
CCND2	6.12E-92	0.910937578
FMR1	1.21E-34	0.908640131
GATM	2.30E-22	0.895633582
NIPA2	7.87E-50	0.894986316
STS	8.81E-46	0.894174304
SPARC	1.21E-37	0.889875824
ACE	2.11E-51	0.879057124
EZH2	6.39E-36	0.870441385
DHCR24	1.41E-52	0.870417114
NPAT	3.99E-74	0.866425058
XPA	2.32E-11	0.865749955
ALDH2	2.28E-47	0.855806877
GPR12	3.62E-63	0.849863818

IGFBP3	5.31E-38	0.844882325
PTGER2	2.22E-51	0.83961072
PAK1	7.36E-26	0.839195349
ITGB3	2.05E-52	0.829204632
HSD17B3	1.57E-65	0.827340673
F5	6.43E-39	0.820180188
AKT2	1.22E-37	0.815406768
LEF1	2.58E-54	0.807101816
CYP11B2	1.19E-23	0.805498277
ITGAV	4.07E-42	0.802213722
CYP7B1	6.71E-61	0.79552417
APC	6.37E-20	0.793158382
PPFIA3	4.39E-32	0.792930479
TP53	1.98E-73	0.79160957
OSBPL8	7.78E-53	0.791509677
ADRB3	7.35E-42	0.788757413
WNT5A	3.17E-47	0.785335861
CDH11	3.07E-33	0.782484118
LIPC	6.40E-35	0.781034625
TCF21	3.45E-21	0.780615993
DPYSL3	4.21E-68	0.771409226
COL1A1	8.54E-41	0.770938539
CD36	8.37E-42	0.765170901
APOD	4.14E-28	0.753485872
INSIG2	2.37E-27	0.750694373
AKT1	1.04E-30	0.749496425
PLCG1	2.77E-79	0.748146587
PPARA	7.33E-38	0.742888127
ISL1	1.71E-44	0.74257211
CDS2	1.55E-47	0.736828252
HDAC1	1.25E-52	0.727869129
HSD17B4	8.56E-37	0.725527856
CD86	2.51E-33	0.723650683
DPYD	7.75E-13	0.72226704
CCL2	3.01E-22	0.718664928
ALOX5	2.30E-49	0.718119627
DUSP9	1.59E-36	0.71787607
PLD1	1.30E-10	0.715322615
PDGFRB	3.32E-11	0.714539446

DNAJB1	3.25E-43	0.710513639
CALM1	4.76E-69	0.710369865
PTGS1	8.81E-28	0.709834889
NFKBIA	4.91E-33	0.707753496
TGFB3	1.26E-22	0.70601823
MGP	2.50E-23	0.704665672
BCL2	3.51E-19	0.701066921

Supplementary Table S2. Cellular component of differentially expressed genes

Cellular component	Gene symbol	Parents Identifier
extracellular region part	CLU, COMT, CTNNB1, DES, EGFR, F3, FASN, ALDH1A3, FGF2, FLNA, CYFIP1, ALOX12, GREM1, GYG1, ANXA1, APLP2, IMPA1, KLK2, LDHB, LOX, MSMB, MYLK, AZGP1, ACTA2, SHH, SOD1, TIMP4, ADIPOQ, COX5A, GREB1, CDH1	GO:0044421
extracellular exosome	CLU, COMT, CTNNB1, DES, F3, FASN, ALDH1A3, FLNA, CYFIP1, ALOX12, GYG1, ANXA1, APLP2, IMPA1, KLK2, LDHB, MYLK, AZGP1, ACTA2, SOD1, ADIPOQ, COX5A, GREB1, CDH1	GO:0070062
extracellular vesicle	CLU, COMT, CTNNB1, DES, F3, FASN, ALDH1A3, FLNA, CYFIP1, ALOX12, GYG1, ANXA1, APLP2, IMPA1, KLK2, LDHB, MYLK, AZGP1, ACTA2, SOD1, ADIPOQ, COX5A, GREB1, CDH1	GO:1903561
extracellular organelle	CLU, COMT, CTNNB1, DES, F3, FASN, ALDH1A3, FLNA, CYFIP1, ALOX12, GYG1, ANXA1, APLP2, IMPA1, KLK2, LDHB, MYLK, AZGP1, ACTA2, SOD1, ADIPOQ, COX5A, GREB1, CDH1	GO:0043230
vesicle	CLU, COMT, CTNNB1, DES, EGFR, F3, FASN, ALDH1A3, FLNA, CYFIP1, ALOX12, GYG1, ANXA1, APLP2, IMPA1, KLK2, LDHB, MYLK, AZGP1, ACTA2, SOD1, SREBF1, ADIPOQ, COX5A, GREB1, CDH1	GO:0031982
membrane raft	CD24, CNR1, CTNNB1, EGFR, LDHB, PTGS2, SHH, CDH1	GO:0045121
membrane microdomain	CD24, CNR1, CTNNB1, EGFR, LDHB, PTGS2, SHH, CDH1	GO:0098857
cytosol	CD24, CDKN1B, CLU, COMT, CTNNB1, DES, FASN, ALDH1A3, FLNA, CYFIP1, FOS, ALOX12, AMD1, GYG1, HIF1A, IMPA1, LDHB, MYLK, OAZ2, ACTA2, SHH, SOD1, SREBF1, SOCS2	GO:0005829
extracellular space	CLU, EGFR, F3, FGF2, GREM1, ANXA1, LOX, MSMB, AZGP1, ACTA2, SHH, SOD1, TIMP4, ADIPOQ	GO:0005615
cytoplasmic part	CD24, CDKN1B, CLU, COMT, CTNNB1, CYP1B1, DES, EGFR, FASN, ALDH1A3, FLNA, CYFIP1, FOS, ALOX12, AMD1, GYG1, ANXA1, HIF1A, APLP2, IMPA1, LDHB, LTC4S, MYLK, OAT, OAZ2, PTGS2, ACTA2, SHH, SOD1, SREBF1, TIMP4, TPD52, SOCS2, ADIPOQ, COX5A, PTGES, CDH1	GO:0044444

Supplementary Table S3. List of predicted signaling pathways of differentially expressed genes in AA men with PCa

**Pathway Names**	**p-value**	**Gene symbol**	**Pathway IDs**
Pathways in cancer	1.60E-12	CDKN1B, CTNNB1, EGFR, AKT1, NFKBIA, PDGFRB, PIK3CA, BCL2, TP53, HIF1A, MMP9, PTGS2, CDH1, APC, FGF2, SMAD3, ITGAV, SHH, ITGAV, WNT5A	5200
Prostate cancer	1.13E-06	CDKN1B, CTNNB1, EGFR, AKT1, AKT2, NFKBIA, NKX3-1, LEF1, PDGFRB, PIK3CA, BCL2, TP53	5215
Focal adhesion	2.95E-05	COL1A1, CTNNB1, EGFR, AKT1, AKT2, FLNA, ITGAV, ITGB3, MYLK, PAK1, PDGFRB, PIK3CA, PXN, BCL2, CCND2	4510
Metabolism of lipids and lipoproteins	3.34E-04	CYP1B1, CYP11B2, DHCR24, FASN, FDFT1, ALOX12, ALOX5, HSD17B3, HSD17B4, LTC4S, STS, INSIG2, PIK3CA, PLA2G2A, PLD1, PPARA, PTGS1, PTGS2, SREBF1, CDS2, CYP7B1, CD36, PTGES	REACT_22258
Arachidonic acid metabolism	3.38E-03	CYP1B1, ALOX12, ALOX5, LTC4S, PTGS1, PTGS2, PTGES	REACT_147851
deactivation of the beta-catenin transactivating complex	4.40E-03	CTNNB1, AKT1, AKT2, HDAC1, APC, LEF1	REACT_264178
Constitutive PI3K/AKT Signaling in cancer	8.65E-03	CDKN1B, EGFR, AKT1, AKT2, FGF2, PDGFRB, PIK3CA, CD86	REACT_147727
Signaling by EGFR	1.16E-02	CDKN1B, EGFR, AKT1, AKT2, FGF2, PDGFRB, PIK3CA, PLCG1, PXN, CALM1, CD86	REACT_9417
Signaling by PDGF	1.90E-02	CDKN1B, EGFR, AKT1, AKT2, FGF2, PDGFRB, PIK3CA, PLAT, PLCG1, CALM1, CD86	REACT_16888
Signaling by FGFR	2.70E-02	CDKN1B, EGFR, AKT1, AKT2, FGF2, PDGFRB, PIK3CA, PLCG1, CALM1, CD86	REACT_9470
Signaling by ERBB2 & DAP12	3.19E-02	CDKN1B, EGFR, AKT1, AKT2, FGF2, PDGFRB, PIK3CA, PLCG1, CALM1, CD86	REACT_115755
MAPK signaling pathway	3.72E-02	DUSP9, EGFR, AKT1, AKT2, FGF2, FLNA, FOS, GADD45B, PAK1, PDGFRB, PLA2G2A, TGFB3, TP53	4010

Supplementary Table S4. Correlation of differentially expressed genes with clinical outcomes in PCa patients

Clinical feature	Gene symbol
Age at diagnosis	AMD1, F3, IGFBP3, APC2, TBXAS1, TYMS, GUCY1A3, CD14, ST6GAL1
Pathology T-stage	KLK2, AZGP1, ALDH1A3, MSMB, SOCS2, GREB1, GREM1, CNR1, IMPA1, ERCC1, PLAT, PXN, MR1, SPARC, EZH2, ALDH2, IGFBP3, F5, CYP7B1, WNT5A, CDH11, PDGFRB, CALM1, TGFBR1, EPHX2, BRCA1, F2R, PTGDS, MEN1, ALOX15, APC2, ABCA5, CDKN2A, FGFR2, BIRC5, SMAD7, KRT5, PTGIR, ALOX15B, GDF15, PDGFB, CYB561, SRD5A2, CTSK, TGFB1
Pathology N-stage	KLK2, AZGP1, GREM1, PXN, FMR1, CYP7B1, TCF21, COL1A1, WNT1, EPHX2, PTGDS, ITGBL1, CDKN2A, PTGIR, SMOX, SFRP4, SRD5A2, IGF1
Residual tumor	SPARC, IGFBP3, CYP7B1, COL1A1, TRAF2, ITPR2, FRZB, BGN, DVL3, HOXB5
Number of lymph nodes	KLK2, AZGP1, MSMB, GREM1, PXN, FMR1, EZH2, CYP7B1, TCF21, WNT1, EPHX2, PTGDS, MEN1 ITGBL1, CDKN2A, PTGIR, PTEN, ALOX15B, SMOX, GSTT1, SFRP4, SRD5A2, SMAD4, PDK4, OXB5, IGF1, INHBA
Gleason Score	KLK2, AZGP1, AMD1, ALDH1A3, TIMP4, NKX3-1, ACTA2, GREB1, MYLK, GREM1, COMT, YP1B1, CDH1, FLNA, LDHB, CDKN1B, PXN, FMR1, EZH2, IGFBP3, CYP7B1, TP53, OSBPL8, WNT5A, CDH11, TRAF2, TGFBR1, BRCA1, F2R, PTGDS, MEN1, ALOX15, APC2, MAOB, CDKN2A, FGFR2, SMAD7, AKAP12, ALOX15B, SMOX, APOE, PIK3R2, PDGFB, FOXF1, CYB561, TRPV6, SRD5A2, SMAD4, ERBB3, IGF1, TGFB1, ST14, TRIM29
PSA level	AZGP1, CTNNB1, PIK3CA, MMP9, NPAT, OSBPL8, MMP12, AR, E2F3, SMOX, INHBA

Supplementary Table S5. List of set of human primer sequence used in quantitative Real-Time PCR analysis.

primer	Forward	Reverse
AMD1	CCCTGTTGAAGCTTGCTAGG	TGGGTACCCTTGGTGAGAAG
ALDH1A3	TCACAGACAACATGC GGATT	TCGGTGCTATTCGCTCTTTT
TPD52	GAGATCAAGCGGAAACTTGG	GAGCCAACAGACGAAAAAGC
OAT	TCGTAAGTGGGGCTATACCG	CTGGTTGGGTCTGTGGAACT
LTC4S	CTTCTCCCTGCAGGTGATCT	GGGAAGTACTCGCTGCAGTT
APPL2	GGGTGTCCCAGCAAGAATTA	GTGACCAGCCCTGTTTTGTT
NKX3.1	GCCAAGAACCTCAAGCTCAC	AGAAGGCCTCCTCTTTCAGG
β-actin	GCACCACACCTTCTACAATGA	GTCATCTTCTCGCGGTTGGC

Supplementary Figure S1. Protein expression of LTC4S, OAT and TPD52 in AA and CA PCa cell lines. Western blot analysis was performed as indicated. Original Western blot membranes representing the designated protein signals, which were identified by either ECL kit (Peirce). GAPDH was used as an internal control.

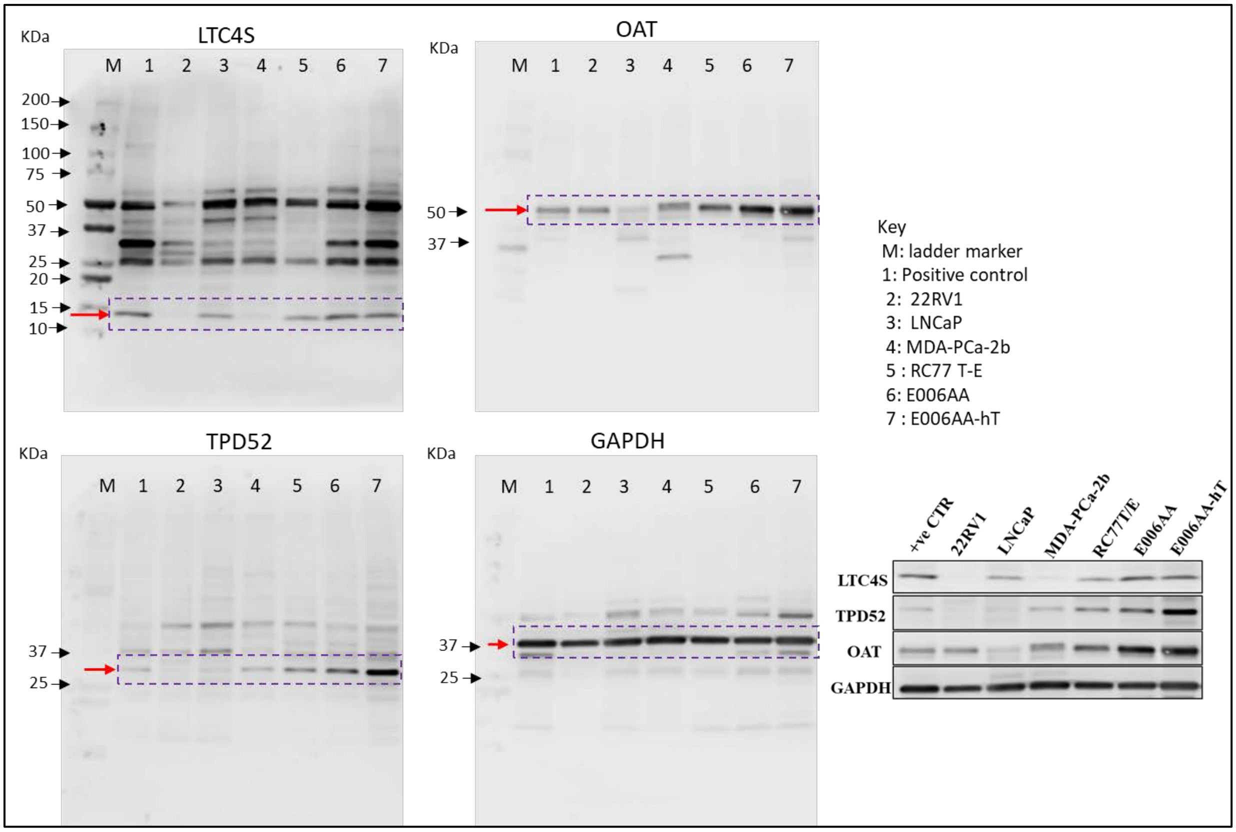

Supplement: Supplementary file 1 — Supplementary information [file 41598_2018_34637_MOESM1_ESM.pdf]
